# Initial Characterization of the Epstein–Barr Virus BSRF1 Gene Product

**DOI:** 10.3390/v11030285

**Published:** 2019-03-21

**Authors:** Yusuke Yanagi, H. M. Abdullah Al Masud, Takahiro Watanabe, Yoshitaka Sato, Fumi Goshima, Hiroshi Kimura, Takayuki Murata

**Affiliations:** 1 Department of Virology, Nagoya University Graduate School of Medicine, Nagoya 466-8550, Japan; yusuke.yanagi@med.nagoya-u.ac.jp (Y.Y.); masud@med.nagoya-u.ac.jp (H.M.A.A.M.); t.nabe.watanabe@gmail.com (T.W.); yssato@med.nagoya-u.ac.jp (Y.S.); fgoshima@med.nagoya-u.ac.jp (F.G.); hkimura@med.nagoya-u.ac.jp (H.K.); 2 Department of Virology and Parasitology, Fujita Health University School of Medicine, Toyoake 470-1192, Japan

**Keywords:** EBV, BSRF1, Golgi apparatus, secondary envelopment

## Abstract

Epstein–Barr virus (EBV) is a ubiquitous virus that causes infectious mononucleosis and several types of cancer, such as Burkitt lymphoma, T/NK-cell lymphoma, and nasopharyngeal carcinoma. As a herpesvirus, it encodes more than 80 genes, many of which have not been characterized. EBV *Bam*HI S rightward reading frame 1 (BSRF1) encodes a tegument protein that, unlike its homologs herpes simplex virus unique long 51 (UL51) and human cytomegalovirus UL71, has not been extensively investigated. To examine the role of BSRF1, we prepared knockout and revertant strains using the bacterial artificial chromosome system. Unexpectedly, the disruption of the gene had little or no effect on EBV lytic replication and the transformation of primary B cells. However, the knockdown of BSRF1 in B95-8 cells decreased progeny production. An immunofluorescence assay revealed that BSRF1 localized to the Golgi apparatus in the cytoplasm, as did its homologs. BSRF1 also associated with *Bam*HI G leftward reading frame 3.5 (BGLF3.5), *Bam*HI B rightward reading frame 2 (BBRF2), and *Bam*HI A leftward reading frame 1 (BALF1), and BALF1 was incorporated into the tegument fraction with BSRF1. Taken together, our results indicate that BSRF1 plays a role in secondary envelopment or virion egress in the cytoplasm, as do its homolog genes.

## 1. Introduction

Epstein–Barr virus (EBV), a herpesvirus, infects more than 90% of the population. In most cases, EBV infection is associated with few or no symptoms, but it can cause infectious mononucleosis, particularly following the primary infection of adolescents [1]. After primary infection, the virus establishes a latent infection in B cells and persists for life. EBV infection can also induce carcinogenesis, including Burkitt lymphoma, Hodgkin lymphoma, T/NK-cell lymphoma, gastric carcinoma, and nasopharyngeal carcinoma [2].

EBV can establish a latent infection [3]. During latency, a limited number of viral genes, such as EBV nuclear antigen 1 (EBNA1) and latent membrane protein 1 (LMP1), are expressed. The virus genome is amplified simultaneously with that of the host in S phase and delivered to daughter cells in the M phase. EBV can also establish lytic infection [4]. The switch from a latent to a lytic infection is termed reactivation. During the lytic cycle, all of the >80 viral genes are expressed, the viral genome undergoes replication, and progeny virus particles are produced [5,6]. The trigger of EBV reactivation in vivo is unclear, but in cell culture, reactivation can be initiated by several reagents, including 12-O-tetradecanoylphorbol-13-acetate (TPA), calcium ionophore, histone deacetylase (HDAC) inhibitors, and transforming growth factor-beta (TGF-β). Otherwise, the exogenous expression of *Bam*HI Z leftward reading frame 1 (BZLF1) (EB1, Zta), a viral immediate–early gene, is sufficient for the reactivation of EBV. Upon the induction of lytic infection, the transcriptional activators encoded by the immediate–early genes induce the expression of early genes. The early genes encode proteins involved in viral DNA replication, such as BALF2 (single-stranded-DNA-binding protein) and BMRF1 (processivity factor of the viral DNA polymerase), thereby initiating viral DNA replication. Late viral genes are transcribed after DNA replication. The late genes encode structural proteins, such as capsid, tegument, and membrane proteins, which contribute to the production of progeny virions. Capsid proteins form an icosahedral structure that encompasses the viral DNA. Glycoproteins, such as glycoprotein B (gB), are embedded in the envelope around the icosahedral nucleocapsid. The tegument is the space between the nucleocapsid and the envelope and is enriched with >10 viral proteins called tegument proteins [7]. The tegument proteins are involved in morphogenesis, envelopment, egress of progeny virions, and during infection, they play a major role in viral ingress and infectivity by modulating the intracellular environment [8]. EBV tegument proteins have not been investigated as extensively as the products of the other viral genes and warrant further research.

The product of the EBV BSRF1 gene is reportedly incorporated into the tegument [9]. Its role in the viral lifecycle is unclear, although those of its homologs in herpes simplex virus (HSV) (UL51) and human cytomegalovirus (HCMV) (UL71) have been established. HSV UL51 is post-translationally modified by phosphorylation [10] and palmitoylation [11] and localized to the cytoplasm, most likely in the Golgi apparatus [11]. It is involved in the morphogenesis, envelopment, and egress of progeny virions [12,13]. HCMV UL71 is also cytoplasmic and is involved in the maturation and envelopment of progeny virions [14,15].

To evaluate the physiological role of BSRF1, we prepared a BSRF1-knockout mutant by introducing a stop codon in the context of EBV-bacterial artificial chromosome (BAC). Unexpectedly, the loss of the BSRF1 gene did not affect viral replication in HEK293 cells. However, the ablation of BSRF1 in B95-8 cells resulted in reduced progeny production. Its localization was predominantly cytoplasmic, and its interactions with other tegument proteins were examined. Together, EBV BSRF1, as with HSV UL51 and/or HCMV UL71, is involved in the egress of virion, although this phenotype could not be observed in HEK293. 

## 2. Materials and Methods

### 2.1. Cell Culture and Reagents

HEK293T, HEK293, and the derivative HEK293 EBV-BAC cells were cultured in Dulbecco’s modified Eagle’s medium (Sigma-Aldrich, St. Louis, MO, USA) containing 10% fetal bovine serum (FBS). Akata(−) and B95-8 cells were cultured in Roswell Park Memorial Institute (RPMI) 1640 medium (Sigma-Aldrich) supplemented with 10% FBS. Peripheral blood mononuclear cells (PBMCs) were collected from healthy adult donors who provided written informed consent, according to protocols approved by the Institutional Review Board of Nagoya University. PBMCs were maintained in RPMI1640 medium (Sigma-Aldrich) supplemented with 10% FBS and nonessential amino acids (Sigma-Aldrich).

Antiserum against BSRF1 protein was obtained by immunizing a rabbit with a synthetic peptide. The polypeptide (NH2-C+RQPEHSGPTELALT-COOH) was synthesized and conjugated to Keyhole Limpet hemocyanin (KLH) by the maleimide method and injected into the rabbits four times. Rabbit antibodies against BZLF1, BALF2, BMRF1, BKRF4, BALF4 (gB), LMP1 and tubulin were reported previously [16,17]. Mouse anti-BMRF1 and -giantin antibodies were obtained from Novocastra (Newcastle, UK) and Abcam (Cambridge, MA, USA), respectively. Anti-FLAG (F3165, F7425) and anti-HA (11583816, 1867423) antibodies were from Sigma-Aldrich. HRP-linked goat antibodies to mouse or rabbit IgG were purchased from Amersham Biosciences (Little Chalfont, UK). Goat anti-rabbit and anti-mouse IgG antibodies conjugated to Alexa 488, 546 and a Zenon mouse IgG labeling kit were from Molecular Probes (Eugene, OR, USA).

### 2.2. Construction of Expression Plasmids

The expression vectors for BZLF1, HA-tagged BSRF1 (pcDNAHABSRF1), HA-tagged BGLF3.5, and HA-tagged BBRF2 were prepared previously [18]. To construct a FLAG-tagged BSRF1 vector, the BSRF1 open reading frame (ORF) together with the FLAG sequence was amplified by PCR using the primers 5′-TAGTCCAGTGTGGTGGATGGCCTTCTATCTCCCAGA-3′ and 5′-AAACGGGCCCTCTAGACTACTTGTCGTCATCGTCTTTGTAGTCCGTTAACGCGAGCTCCGTGG-3′, and inserted into the pcDNA3 vector (Invitrogen, Carlsbad, CA, USA) using the In-Fusion cloning system (Takara Bio, Kusatsu, Japan). FLAG and HA were tagged at the C-terminus because the N-terminal region may be involved in palmitoylation.

### 2.3. Construction of BSRF1-KO EBV-BAC and Cloning of HEK293 Cells Maintaining EBV-BAC

EBV-BAC DNA (B95-8) was a gift from W. Hammerschmidt [19]. For mutagenesis, the EBV-BAC genome was manipulated in Escherichia coli as described previously [17]. First, a DNA fragment containing the Neo/St (neomycin-resistant and streptomycin-sensitive genes) cassette flanked by the EBV BSRF1 sequence was prepared by PCR using the primers 5′-CCAGGAATAGATACAGCCAGCTCCCTGAGGAGCCGGAGACCTTTGAGTGCCCGGACCGCTGGCCTGGTGATGATGGCGGGATC-3′ and 5′-TTTCTTAGCAAATCTCCCACCTGCACACCAGGGGGCAGGCCCAGATCTATCTCGGCTCGCTCAGAAGAACTCGTCAAGAAGG-3′. To insert the Neo/St cassette into the BSRF1 gene, the DNA fragment was electroporated into bacteria harboring EBV-BAC, and Red/ET recombination was induced. Kanamycin-resistant colonies were selected and successful recombination was confirmed. The cassette was next excised by replacing with a BSRF1 sequence containing a stop codon. A stop codon was first introduced into the plasmid vector for HA-tagged BSRF1 by a reverse PCR method using the primers 5′-AGCGAGCCGAGATAGATCTGG-3′ and 5′-AGCGGTCCGGGCACTCAAAG-3′. Subsequently, the BSRF1 sequence with a stop mutation was amplified by PCR and used to replace the cassette in EBV-BAC. After replacement, bacteria with dBSRF1-stop EBV-BAC were selected using streptomycin. Likewise, the revertant strain (dBSRF1-stop/R) was constructed by inserting and replacing the Neo/St cassette with the wild-type BSRF1 sequence. Recombination was confirmed by PCR, sequence analysis, and electrophoresis of the BamHI- or EcoRI-digested viral genome.

HEK293 cells were transfected with recombinant EBV-BAC DNA using Lipofectamine 2000 reagent, and cultured 10 cm dishes maintained with 150 μg/mL hygromycin B. After 2 weeks, green fluorescent protein (GFP)-positive, hygromycin-resistant cell colonies were cloned.

### 2.4. Transfection, Immunoblotting, Viral DNA Quantification, and Progeny Titration

For lytic induction, HEK293 EBV-BAC cells were transfected with the BZLF1 expression plasmid by electroporation using the Neon Transfection System (Thermo Fisher Scientific, Waltham, MA, USA).

Immunoblotting was carried out as described previously [17]. Two days after transfection, the cells were washed with PBS, harvested in tubes, and solubilized in sample buffer by sonication. After heat treatment, the samples were subjected to sodium dodecyl sulfate-polyacrylamide electrophoresis (SDS-PAGE), then transferred to a polyvinylidene fluoride membrane (Immobilon-P, Millipore, Burlington, MA, USA). The membrane was blocked in skim milk, washed with PBS containing 0.05% Tween-20 (PBST), and treated with the primary antibody (anti-BZLF1, -BALF2, -BMRF1, -BKRF4, -BALF4, -BSRF1, or -tubulin antibody) diluted in Solution 1 of Can Get Signal (TOYOBO, Osaka, Japan). Next, the membrane was washed thrice with PBST, incubated with the secondary antibody diluted in Solution 2 of Can Get Signal, and washed extensively with PBST. After soaking into Luminata Western HRP Substrate (Millipore), images were captured using an Ez-CaptureMG (ATTO).

The EBV DNA level in HEK293 EBV-BAC cells was quantified using quantitative PCR (qPCR) as described previously [20]. Briefly, at 2 days after BZLF1 transfection, the cells were washed with PBS, lysed using a detergent and sonication, and treated with proteinase K. The samples were directly subjected to qPCR assays after heat-inactivation of proteinase K. qPCR was performed using the Fast Start Universal Probe Master (Rox; Roche Applied Science, Penzberg, Germany). A calibration curve was generated from the values of serially diluted samples. The relative cell-associated viral DNA level was normalized to that of host genomic DNA. The primers used were published previously [20].

The EBV DNA level in culture medium was quantified by qPCR as above, except that DNA samples were collected from culture medium treated with DNase I before DNA extraction to eliminate naked viral DNA, as described previously [16]. DNA was purified using a DNease Blood and Tissue Kit (Qiagen, Hilden, Germany).

For titration, virus solutions were mixed with Akata(−) cells, rotated at room temperature for 3 h, centrifuged, and the supernatants decanted. The cell pellets were mixed with fresh medium and cultured for 2 days; infected cells were those positive for GFP. The cells were fixed in 1% formaldehyde, washed with PBS, and the GFP-positive cell ratio was assessed using a FACS Calibur G5 (Becton Dickinson).

### 2.5. Transformation Assay

Serial 10-fold dilutions of normalized virus solutions were infected with 1 × 10^5^ PBMCs in a 96-well plate in the presence of cyclosporine A. The medium was changed every 5 days. The number of transformed wells was counted after 3 weeks of incubation to calculate the 50% transforming dose.

### 2.6. Knockdown of BSRF1 by siRNA

Control or either of the two different siRNAs was transfected into B95-8 cells by electroporation using the Neon Transfection System (Thermo Fisher Scientific). The siRNA sequences used are as follows; siControl, 5′-GCAGAGCUGGUUUAGUGAAtt-3′ and 5′-UUCACUAAACCAGCUCUGCtt-3′; siBSRF1-1, 5′-GCCAAUAGCAUCACGGAUCtt-3′ and 5′-GAUCCGUGAUGCUAUUGGCtt-3′; siBSRF1-2, 5′-GCGGCAUCGAGAUGGACGAtt-3′ and 5′-UCGUCCAUCUCGAUGCCGCtt-3′. After 3 days, protein samples were collected for immunoblotting. The progeny virus was harvested at 6 days after transfection for titration by immunofluorescence. 

### 2.7. Immunofluorescence Analysis

HEK293 EBV-BAC cells were transfected with the indicated expression vectors for 2 days, fixed with 70% ethanol, and permeabilized with 0.1% Triton X-100. The samples were next blocked with 10% normal goat serum (Funakoshi) and incubated with the primary antibodies (anti-actin, -HA, -BMRF1, and -giantin). After washing, the cells were treated with the secondary antibodies, washed, and mounted with ProLong Gold antifade reagent containing 4′,6-diamidino-2-phenylindole (DAPI; Molecular Probes). The samples were analyzed using an LSM880 confocal microscope (Zeiss).

### 2.8. Immunoprecipitation

Immunoprecipitation (IP) was carried out as described previously [16]. Briefly, HEK293T cells transfected by lipofection with the indicated expression vectors were solubilized in IP lysis buffer. The lysates were mixed with an anti-FLAG or -HA antibody and Protein G Sepharose (GE Healthcare, Chicago, IL, USA), washed in IP lysis buffer and the precipitates were lysed in sample buffer for immunoblotting. The samples were resolved by SDS-PAGE, blotted, and immunoblotted using an anti-FLAG or -HA antibody.

### 2.9. Fractionation of Extracellular Virions

Culture supernatants of EBV B95-8 cells transfected with the indicated expression plasmids were collected after 5 days. After low-speed centrifugation and filtration, the supernatants were mock-treated, treated with 1% NP-40, treated with 1% NP-40 plus 300 mM NaCl, and subjected to ultracentrifugation (himac CP 60E, Hitachi, Tokyo, Japan) at 15,000 rpm for 1 h. The pellets were solubilized in sample buffer and resolved by SDS-PAGE, followed by immunoblotting using anti-FLAG, -HA, -gB, -BRRF2, -BKRF4, and -tubulin antibodies.

## 3. Results

### 3.1. Preparation of BSRF1-Knockout EBV

**Figure 1:**
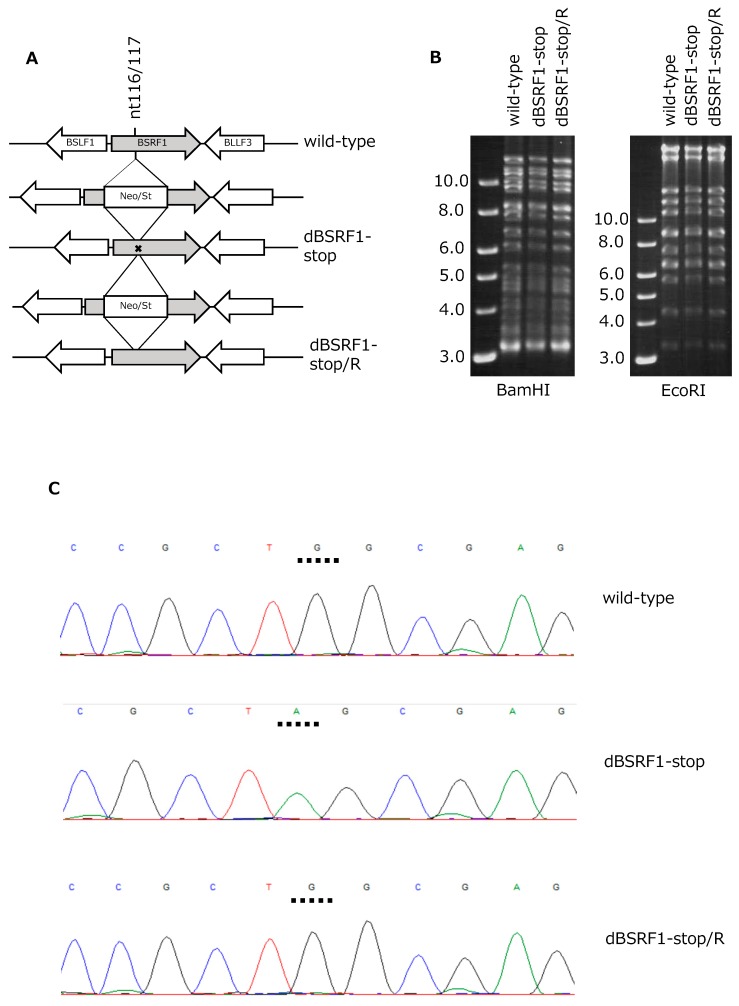
Construction of the *Bam*HI S rightward reading frame 1 (BSRF1) knockout (KO) mutant. (**A**) Schematic representation of the recombination of the Epstein–Barr virus (EBV)-bacterial artificial chromosome (BAC) genome using neomycin- and streptomycin-sensitivity genes in tandem (Neo/St). The insertion mutant was produced by inserting a Neo/St cassette at nucleotides 116 and 117 of the BSRF1 gene. To construct the KO virus (dBSRF1-stop), the cassette was replaced by the BSRF1 sequence with a stop codon. The same cassette was again inserted to prepare the intermediate, and replaced with the wild-type BSRF1 sequence to generate the repaired strain, dBSRF1-stop/R. (**B**) Agarose gel electrophoresis of recombinant EBV-BAC DNA treated with BamHI and EcoRI. (**C**) Sequence data of the recombinant EBVs.

Because no BSRF1-KO mutant of EBV has been reported, we knocked out the BSRF1 gene in the context of EBV-BAC to analyze its role in the EBV lifecycle. A Neo/St cassette was first inserted between nucleotides 116 and 117 of the BSRF1 gene and replaced with the BSRF1 sequence with a stop codon to prepare the KO mutant (dBSRF1-stop) (Figure 1A). The stop codon (TAG) was introduced at Trp 39 (TGG) of BSRF1. A revertant strain (dBSRF1-stop/R) was prepared by inserting and removing the Neo/St cassette (Figure 1A).

To confirm the integrity of the viral genome, recombinant EBV-BAC DNA was digested with BamHI or EcoRI, followed by electrophoresis in an agarose gel (Figure 1B). The identical band patterns of the wild-type, dBSRF1-stop, and dBSRF1-stop/R indicate that no obvious deletions or insertions were present in the EBV-BACs. Sequence analysis confirmed the presence of the intended mutation (Figure 1C).

We transfected HEK293 cells with the wild-type, dBSRF1-stop, and dBSRF1-stop/R EBV-BAC DNAs, followed by hygromycin selection. This resulted in the isolation of GFP-positive, hygromycin-resistant cell lines, in which the recombinant virus was latently maintained as an episome, for each EBV-BAC strain.

### 3.2. BSRF1 Is Not Required for EBV Replication in HEK293 Cells

**Figure 2:**
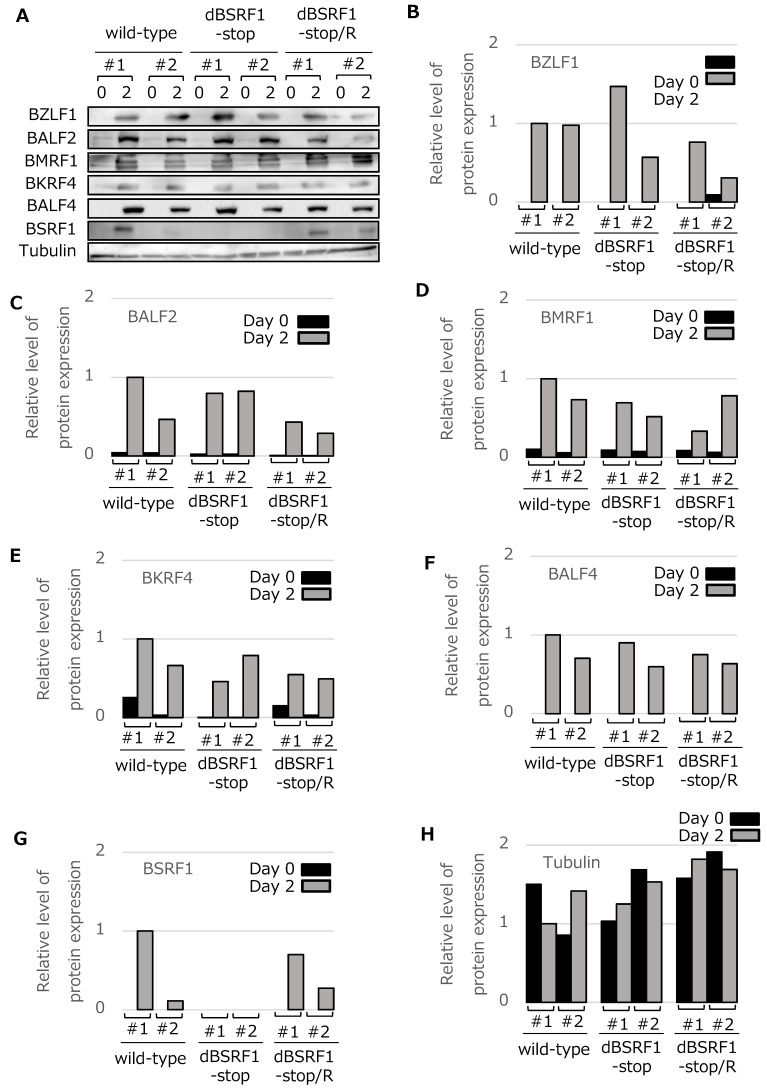

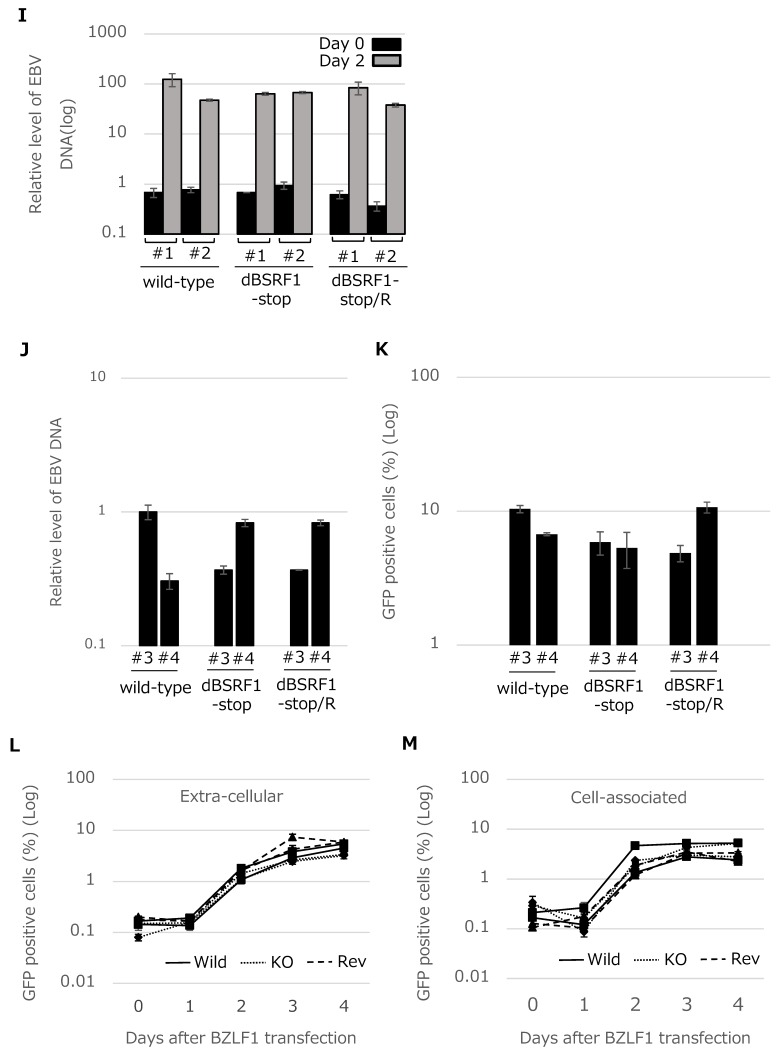
Protein expression, DNA replication, and progeny production of BSRF1 KO EBV in HEK293 cells. (**A**) Viral protein expression in HEK293. HEK293 EBV-BAC cells were transfected with the BZLF1 expression plasmid by electroporation. Cells were harvested after 2 days and subjected to immunoblotting with the indicated antibodies. (**B**–**H**) Quantitative data. Band intensity was quantified using ImageJ software. (**I**) Viral genome DNA replication in HEK293. Cells transfected as in (A) were harvested after 2 days and subjected to qPCR for EBV and host cell genomic DNA. The means ± SD of three independent biological replicates are shown after normalization to the value of the host control. (**J**) Production of progeny into the culture media. Cells transfected as in (A) were cultured for 3 days, and the medium was collected after centrifugation, treated with DNase, and subjected to DNA extraction and quantification by qPCR. The means ± SD of three independent biological replicates are shown. (**K**) Infectivity of the progeny in the culture media. The same medium as in Figure 2J was used to infect Akata(−) cells. The percentage of green fluorescent protein (GFP)-positive cells was determined by fluorescence-activated cell sorting (FACS) analysis and is shown as logarithmic values. The means ± SD of three independent biological replicates are shown. (**L**, **M**) Time course and comparison of extracellular- and cell-associated progeny levels. Cells were transfected as in (A) with the BZLF1 expression vector, and the medium and cells were separately harvested at the indicated time points for titration using Akata(−) cells. The means ± SD of three independent biological replicates are shown. Cell-clone numbers are also shown (#1, #2, #3, #4).

We analyzed whether the BSRF1 KO strain exhibited defects in lytic replication (Figure 2). The HEK293 EBV-BAC cells prepared above were transfected with a BZLF1 expression vector and harvested for immunoblotting on days 0 and 2 (Figure 2A–H; B–H show quantitative values). We analyzed two representative clones per strain. BSRF1 expression was not detected in the dBSRF1-stop cells, whereas the protein was produced in wild-type and revertant cells. BSRF1 production was weaker in one of the wild-type cells for unknown reasons (Figure 2A,G). The production of viral immediate–early (BZLF1), early (BALF2 and BMRF1), and late (BKRF4 and BALF4) proteins was induced and was not affected by the disruption of the BSRF1 gene (Figure 2A–H). 

Viral DNA levels in cells after lytic induction were quantified by qPCR and normalized to the host genomic DNA level (Figure 2I). The viral DNA level was increased about 100-fold by lytic induction in all strains, indicating that the BSRF1 gene is not required for viral DNA replication (Figure 2I).

We next assayed progeny virions in the culture medium. First, viral genomic DNA in virions was quantitated by qPCR. The virion levels in medium were not decreased by KO of the BSRF1 gene (Figure 2J). We assessed the infectivity of the progeny virions in Akata(−) cells. Akata(−) cells infected with the recombinant viruses are GFP-positive. The GFP-positive ratio of the BSRF1 KO was similar to that of the wild-type and revertant strains at 3 days (Figure 2K). We also assessed the viral titer daily, and collected cell-associated and non-cell-associated virion samples. The disruption of the BSRF1 gene did not affect the production of cell-free or cell-associated infectious progeny virions (Figure 2L,M).

### 3.3. BSRF1 Is Not Required for B-Cell Transformation

**Figure 3:**
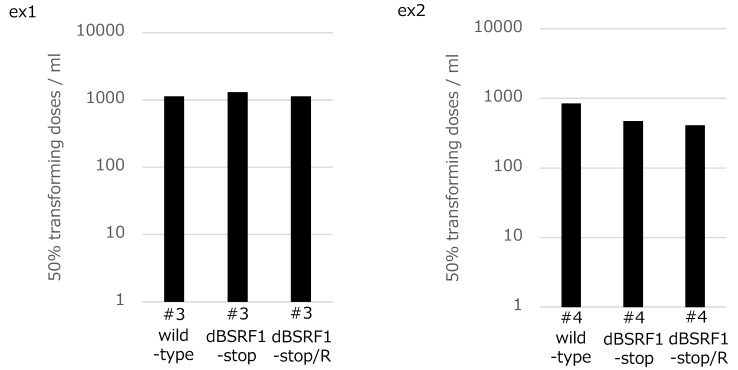
Transformation efficiency of BSRF1 KO mutant virus. Viruses in culture medium of HEK293 EBV-BAC cells were prepared and the titers were determined using Akata(−) cells. After normalization of the titers, 10-fold serial dilutions were prepared and used to separately infect peripheral blood mononuclear cells (PBMCs) from two healthy donors (**ex1** and **ex2**). Bars indicate 50% TU/mL values. Cell-clone numbers are also shown (#3, #4).

EBV can immortalize primary B cells. To determine whether the BSRF1 gene product is involved in this process, PBMCs from two donors were separately infected with the indicated virus strains after titer normalization. The transformation efficiency of the KO virus was comparable to that of the wild-type and revertant viruses (Figure 3), indicating that the BSRF1 gene is not required for B-cell transformation.

### 3.4. BSRF1 Is Required for Efficient Progeny Production in B95-8 Cells

**Figure 4:**
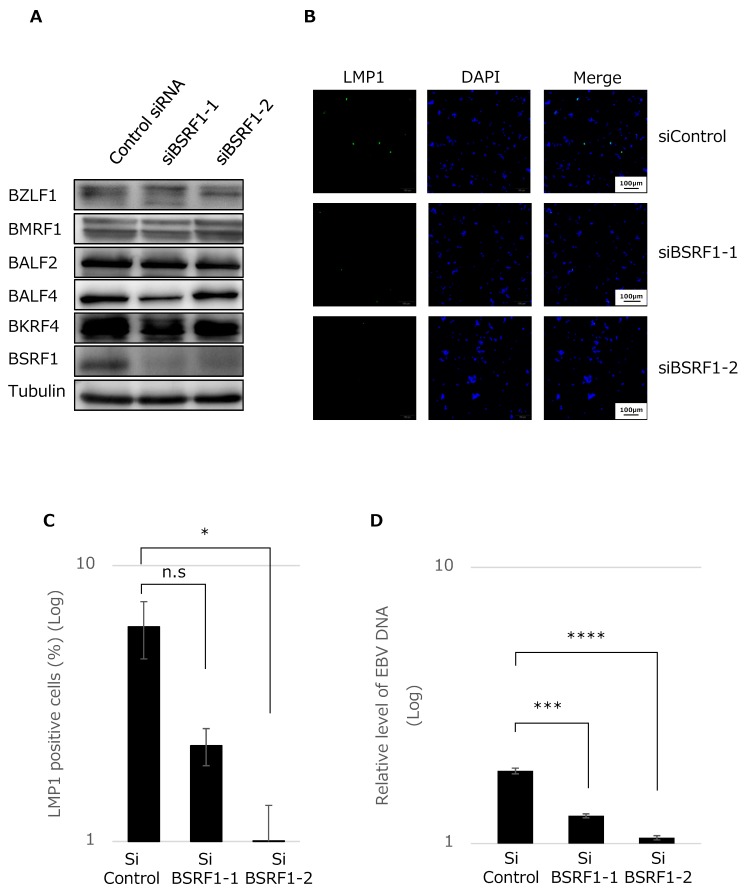
Knockdown of BSRF1 decreased progeny production. (**A**) Effect of BSRF1 knockdown on viral lytic protein expression in B95-8 cells. B95-8 cells were transfected by electroporation with two independent siRNAs (siBSRF1-1 and -2) or control siRNA and incubated. Cells were harvested after 3 days and subjected to immunoblotting with the indicated antibodies. (**B**,**C**) Infectivity of the progeny virus particles produced from B95-8. Six days after the transfection of siRNA as in (A), progeny virus produced into the media was inoculated with Akata(−) cells for 2 days, followed by immunofluorescence assay by using anti-LMP1 antibody (B). Percentage of green fluorescence-positive cells over total cells is shown in (C). Production of progeny in the culture media. B95-8 cells were transfected and cultured as in (B). (**D**) The medium was collected after centrifugation, treated with DNase, and subjected to DNA extraction and quantification by qPCR. The means ± SD of three independent biological replicates are shown. n.s, not significant.; *, *P* < 0.05; ***, *P* < 0.0001.

Because the disruption of the BSRF1 gene did not cause any phenotypic difference in HEK293 cells, we next used B95-8 cells. The expression of the BSRF1 gene in B95-8 cells was knocked down by siRNA. A successful reduction in BSRF1 protein levels was observed in samples treated with siBSRF1-1 or 2, while the production of other viral proteins appeared normal (Figure 4A). 

After knockdown, the progeny virus produced in the medium was harvested from the supernatant, and the infectivity of the progeny was examined in Akata(−) cells. We here stained infected cells by immunofluorescence using anti-LMP1 antibody. As shown in Figure 4B,C, the knockdown of BSRF1 resulted in decreased infectivity. 

Then, viral genomic DNA in virions in the culture medium was measured after DNase treatment by qPCR, as in Figure 2J. Interestingly, the ablation of BSRF1 gene significantly reduced progeny virion DNA levels (Figure 4D). Therefore, it is suggested that BSRF1 plays a role in progeny production, although the expressions of viral genes including late genes were not affected by BSRF1 knockdown. 

### 3.5. Cytoplasmic Localization of BSRF1 Protein

**Figure 5:**
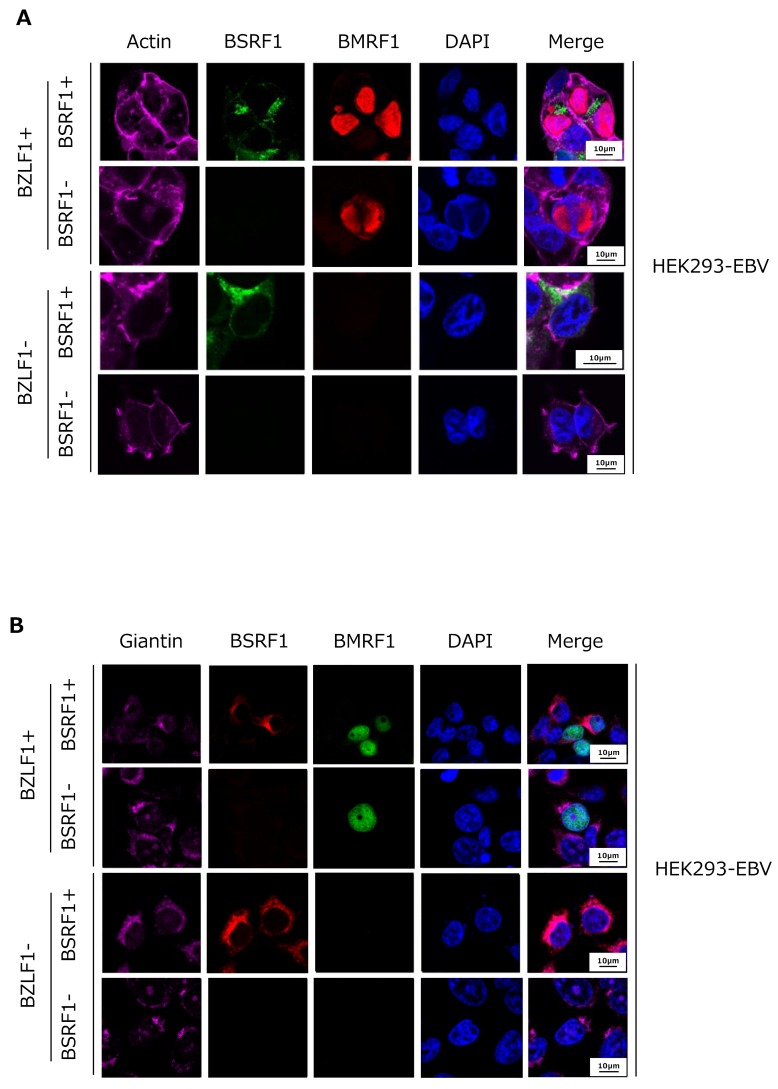
Subcellular localization of BSRF1. (**A**) HEK293 EBV-BAC (wild-type) cells were transfected by lipofection with the expression vectors indicated at left. After 2 days the cells were fixed, stained with phalloidin (actin, purple), an anti-HA (BSRF1, green) antibody, an anti-BMRF1 (red) antibody, and DAPI (blue), then visualized using a confocal laser microscope. (**B**) HEK293 EBV-BAC (wild-type) cells were transfected likewise, and stained with an anti-giantin (Golgi apparatus, purple) antibody, an anti-HA (BSRF1, green) antibody, an anti-BMRF1 (red) antibody, and DAPI (blue).

We next investigated the intracellular localization of BSRF1 in HEK293 EBV-BAC cells by immunofluorescence (Figure 5). An initial experiment using our antiserum against BSRF1 failed due to its weak reactivity. Therefore, we transfected cells with an HA-tagged BSRF1 expression vector and stained with an anti-HA antibody (green). Actin (purple), BMRF1 protein (red), and DAPI (blue) staining were also performed. In the cells transfected with the BZLF1 expression vector (BZLF1+), BMRF1 accumulated in the nucleus and formed replication compartments, whereas no red staining was observed in the absence of the BZLF1 vector (Figure 5A). HA-tagged BSRF1 protein localized predominantly in the cytoplasm, likely in the Golgi apparatus, irrespective of lytic induction by BZLF1 transfection (Figure 5A).

Next, we co-stained with a Golgi apparatus marker, giantin [21] (Figure 5B, purple). BSRF1 protein co-localized with the Golgi marker, with or without lytic induction by BZLF1 (Figure 5B). This localization pattern of BSRF1 protein is in agreement with that of its homologs, HSV UL51 and HCMV UL71 [11,14].

### 3.6. Association of BSRF1 with Other Viral Proteins

**Figure 6:**
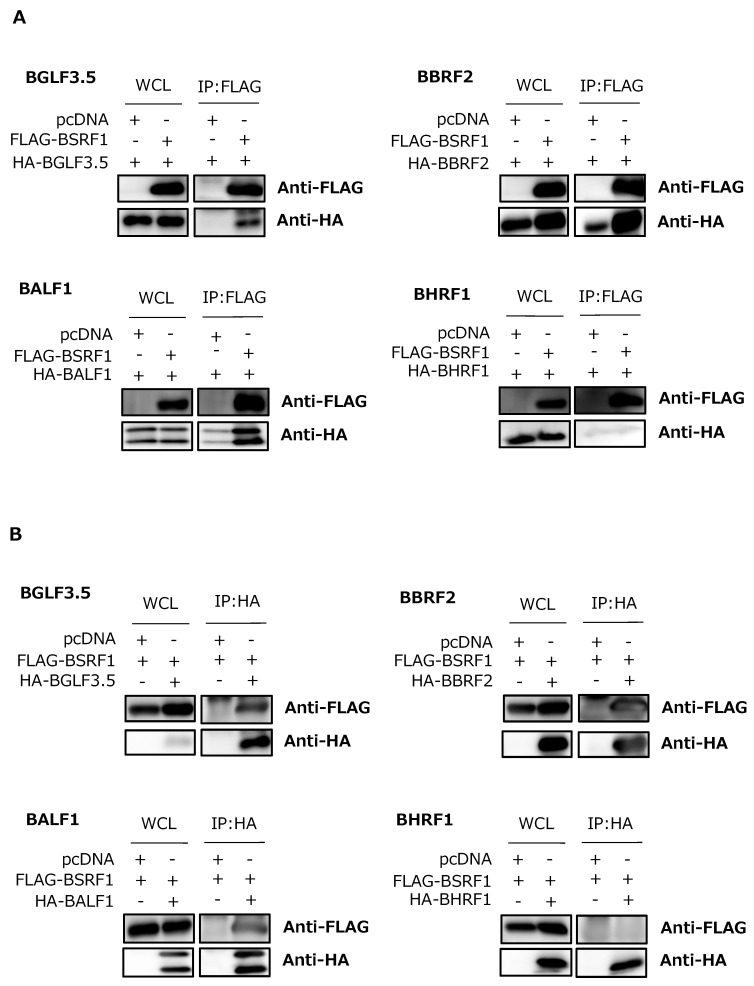

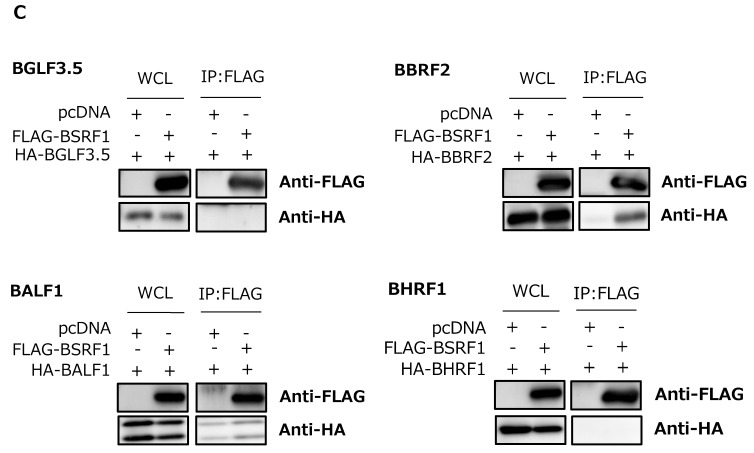
Association of BSRF1 with other viral proteins. (**A**) HEK293T cells were cotransfected by lipofection with the indicated expression vectors. After 24 h, whole-cell lysates were prepared and a portion was subjected to immunoblotting using anti-FLAG and -HA antibodies. The remaining lysates were subjected to immunoprecipitation (IP) using an anti-FLAG antibody, followed by immunoblotting with anti-FLAG and -HA antibodies. (**B**) The lysates were immunoprecipitated with an anti-HA antibody, followed by immunoblotting. (**C**) HEK293 EBV-BAC (wild-type) cells were transfected by lipofection with the indicated vectors, and subjected to IP using an anti-FLAG antibody followed by immunoblotting.

Although we did not determine the physiological role of BSRF1 in HEK293 cells (Figure 2), knockdown experiment and immunofluorescence assays showed that BSRF1 has similarity to HSV UL51 and HCMV UL71 (Figure 4 and Figure 5). Therefore, we examined whether BSRF1 shares other features with its homologs in other herpesviruses. Because HSV UL51 interacts with UL7 [13] and UL14 [22]—homologs of BBRF2 and BGLF3.5, respectively—we used these in the experiment. IP assays showed that the HA-tagged BGLF3.5 and BBRF2 gene products co-precipitated with FLAG-tagged BSRF1 (Figure 6A). Next, we examined the interactions between BSRF1 and EBV vBcl2 proteins, as Kaposi’s sarcoma-associated herpesvirus (KSHV) ORF55, a homolog of BSRF1, precipitated with ORF16, the vBcl2 of KSHV [23]. EBV encodes two vBcl2s, BALF1 and BHRF1 [7], and thus we tested both. BSRF1 precipitated with BALF1 but not BHRF1 (Figure 6A).

We next repeated the IP experiment using an anti-HA antibody (Figure 6B). Regardless of the antibody for precipitation, BSRF1 associated with BGLF3.5, BBRF2, and BALF1, but not with BHRF1 (Figure 6B).

Whether the above interactions occur in infected cells was next evaluated (Figure 6C). Interestingly, the presence of other viral proteins inhibited the BSRF1-BGLF3.5 interaction, and partly reduced that of BSRF1-BALF1; in contrast, the BSRF1-BBRF2 association was unaffected. Therefore, BSRF1 interacts with BBRF2, and other EBV proteins might somehow inhibit the interaction of BSRF1 with BGLF3.5 and BALF1.

### 3.7. BSRF1 Was Present in Virions with BALF1

**Figure 7:**
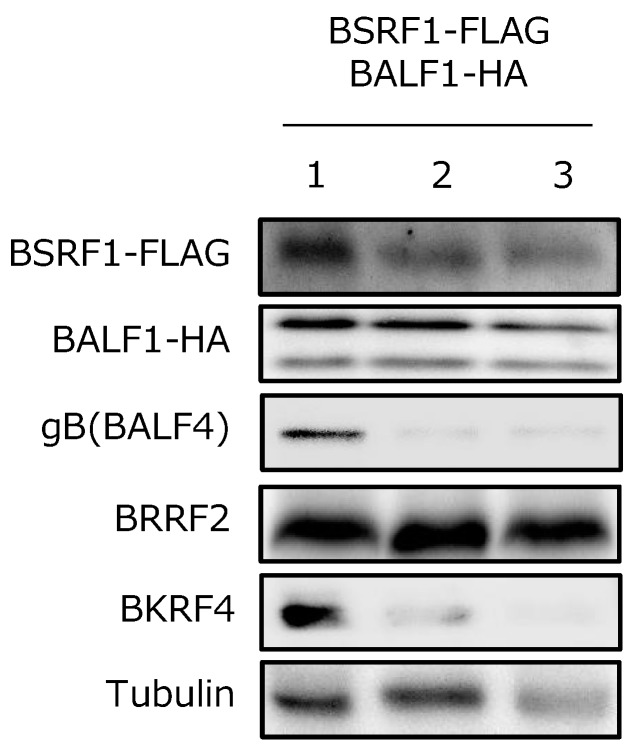
Incorporation of BSRF1 and BALF1 in virions. EBV particles were prepared from B95-8 cell culture medium and transfected with the indicated expression vectors. The medium was precleared via low-speed centrifugation and filtration. Virions were collected by ultracentrifugation in the absence (lane 1) or presence of NP-40 (lane 2) or NP-40 plus 300 mM NaCl (lane 3), followed by immunoblotting with the indicated antibodies.

KSHV ORF16 interacts with ORF55; both are incorporated into KSHV virions and play a role in virion morphogenesis [23]. Therefore, we investigated whether BSRF1 and BALF1 were present in purified virions (Figure 7). To this end, B95-8 cells were transfected with expression plasmids for FLAG-tagged BSRF1 and HA-tagged BALF1 for 5 days, and virions in the supernatant were collected by ultracentrifugation in the absence (lane 1) or presence of 1% NP-40 (Figure 7, lane 2) or 1% NP-40 and 300 mM NaCl (lane 3). The glycoprotein (gB) and a tegument protein (BKRF4) were detected in the virion (lane 1), but were removed from the virion by detergent treatment (lane 2) [16]. Two other tegument components, tubulin and BRRF2 [9,16], were tightly attached to the nucleocapsids and were not dissociated by treatment with a detergent and salt, although tubulin may have been slightly dissociated (lane 3). BSRF1 and BALF1 bound tightly to the nucleocapsid. Thus, as with its KSHV homolog, BSRF1 interacts with BALF1 and is incorporated into virions.

## 4. Discussion

BSRF1 is one of the tegument proteins of EBV [9] and is conserved among herpesviruses. First, we analyzed the role of BSRF1 protein by generating a KO virus using the BAC system. BSRF1 was not required for lytic replication; i.e., it was dispensable for the expression of viral genes, viral DNA synthesis, and production of progeny virions in HEK293 cells (Figure 2). Moreover, the BSRF1 gene did not play a role in the immortalization of peripheral B cells (Figure 3). This was unexpected because the disruption of the HSV UL51 gene reduces virion production by one or two orders of magnitude [10,12] and by 16-fold in an HCMV UL71 mutant [15]. Moreover, ORF55 of murine gammaherpesvirus-68 is essential for viral replication [24]. We do not know why EBV BSRF1 was dispensable in HEK293, but speculate that cell type may account for this discrepancy. Indeed, the phenotype resulting from the KO of some EBV genes, such as BGLF3.5 (homolog of HSV UL14) and BRRF1 (gammaherpesvirus specific), was not observed in our HEK293 system [25,26]. Because both HSV UL51 and UL14 promote the secondary envelopment of nucleocapsids, a cellular factor that inhibits viral secondary envelopment might be absent or inactive in HEK293 cells. Otherwise, HEK293 cells might possess a factor that supports secondary envelopment in the absence of viral gene products. Taking such possibilities into account, we infected other cell lines with BSRF1-KO EBV. Recombinant EBV-BAC (B95-8) virus stably infected Akata(−) and AGS cells, but we could not induce the lytic cycle and so failed to observe the role of BSRF1. Because EBV Akata can stably infect and induce the lytic cycle in Akata(−) and AGS cells (16), we attempted to produce KO Akata virus using the CRISPR/Cas9 system, but were unsuccessful.

To circumvent this limitation, we next carried out a knockdown experiment by using the B95-8 cell line (Figure 4). When the expression of BSRF1 gene was successfully inhibited, the production of virus progeny in the medium was notably reduced. This result suggests that EBV BSRF1 has similar functions as HSV UL51 or HCMV UL71 [10,12,15]. 

BSRF1 protein localized predominantly in the cytoplasm, most likely in the Golgi apparatus, when singly expressed or expressed with other viral proteins during virus replication (Figure 5). This result is in agreement with previous reports on homologs of BSRF1 [11,14]. Moreover, BSRF1, as with its homologs, interacted with BBRF2, BGLF3.5, and BALF1 under overexpression conditions (Figure 6A,B). It is interesting that the interaction of BSRF1 with BGLF3.5 and BALF1 was inhibited in infected cells (Figure 6C). Because HSV UL51 binds UL14 [22] and KSHV ORF55 interacts with ORF16 [23] in infected cells, this inhibition is likely specific to EBV.

Because BALF1 encodes one of the vBcl2 proteins of EBV [27], and BALF1 was incorporated into virion particles (Figure 7), BALF1 protein may play a role in the immortalization of primary B cells upon de novo infection; vBcl2 protein might be released into the cytoplasm after infection and prevent apoptosis. However, the disruption of the BSRF1 gene did not impair immortalization (Figure 3). 

The knockdown experiment of BSRF1 (Figure 4), intracellular localization (Figure 5), protein–protein interactions (Figure 6), and virion incorporation data (Figure 7), together with the known functions of its homologs in other herpesviruses, strongly suggest that BSRF1 is involved in secondary envelopment, maturation, or egress of progeny virion. We report here that the EBV BSRF1 gene is not essential for viral replication in HEK293 cells, but plays a similar role to its homologs in other herpesviruses in B95-8 cells. Further research on the function of the EBV BSRF1 gene is thus warranted.
